# Multisystem Radiologic Manifestations of Erdheim-Chester Disease

**DOI:** 10.1155/2016/2670495

**Published:** 2016-05-31

**Authors:** Umairullah Lodhi, Uzair Sarmast, Saadullah Khan, Kavitha Yaddanapudi

**Affiliations:** Department of Radiology, Stony Brook University Hospital, 101 Nicolls Road, Stony Brook, NY 11794, USA

## Abstract

Erdheim-Chester Disease is a rare form of multiorgan non-Langerhans' cell histiocytosis that affects individuals between the ages of 50 and 70 with an equal distribution among males and females. It is associated with significant morbidity and mortality that is mostly due to infiltration of critical organs. Some of the sites that Erdheim-Chester Disease affects include the skeletal system, central nervous system, cardiovascular system, lungs, kidneys (retroperitoneum), and skin. The most common presenting symptom of Erdheim-Chester Disease is bone pain although a large majority of patients are diagnosed incidentally during a workup for a different disease process. Diagnosing Erdheim-Chester Disease is challenging due its rarity and mimicry to other infiltrative processes. Therefore, a multimodality diagnostic approach is employed with imaging being at the forefront. As of date, a comprehensive radiologic review of the manifestations of Erdheim-Chester Disease has rarely been reported. Here we present radiologic findings of an individual suffering from Erdheim-Chester Disease.

## 1. Introduction

Erdheim-Chester Disease (ECD) is a rare form of non-Langerhan cell histiocytosis that was first described by Chester in 1930 [1]. Since then about 550 cases have been reported in the medical literature [2]. The characteristic features of this disease are related to the multiorgan tissue infiltration of lipid-laden macrophages, multinucleated giant cells, and inflammatory cells composed of lymphocytes and histiocytes [3]. Clinical manifestations of ECD vary among individuals, ranging from an indolent focal disease to a life threatening organ failure [4]. Therefore, prompt diagnosis of this disease is paramount for a favorable outcome. ECD primarily affects adults who are between their 5th and 7th decades of life [5]; however cases have been reported in patients between 7 and 84 years [6]. The etiology of this disease is largely unknown [7] and the presenting symptoms are also often nonspecific [6] which add hindrance in accurately diagnosing this disease in a timely fashion.

Therefore, diagnosis of ECD relies largely on radiologic evidence leading to histologic confirmation. Previously, findings have been described on radiographs, 99mTc bone scintigraphs, computed tomography (CT), and magnetic resonance (MR) imaging scans that could clue one into diagnosing ECD. For example, on conventional radiographs of the long bones, bilateral cortical sclerosis involving the diametaphyseal regions is commonly seen in ECD [8]. Abnormally strong uptake of radioactive tracer at the distal ends of the long bones as observed on 99mTc bone scintigraphs is also noted. Either of these findings could lead to tissue sampling of the lesions for histologic analysis. The histological diagnosis is met when typical ECD histiocytes are found in the examined lesion while testing positive for CD68, CD163, and Factor XIIIa and negative for CD1a on immunohistochemical staining [2, 9]. As of date, a comprehensive review of the radiologic manifestation of ECD with pathognomonic features such as “hairy kidney” and “coated aorta” especially when it occurs in the same individual has rarely been reported. These findings are described and illustrated along with various other radiologic manifestations with the goal of helping physicians in accurately diagnosing this disease.

## 2. Case Report

This patient is a 35-year-old male with no significant past medical history who presented to the emergency department with symptoms of “redness and swelling in his eyes associated with purulent discharge” that had failed to improve on outpatient antibiotic regimen. On further history, patient revealed that for about a year and a half, he had been experiencing grittiness and a “bulging” feeling in his eyes that he thought were due to seasonal allergies as well as loss of balance when standing or walking. His ophthalmologist had ordered an orbital CT scan that revealed retroorbital soft tissue masses. An outpatient biopsy of these masses was nondiagnostic and the patient was subsequently admitted for further evaluation. On physical exam, the patient had bilateral erythematous conjunctiva associated with exophthalmoses without any evidence of lid lag or thyromegaly. Cardiovascular and pulmonary examinations were normal. There was no hepatosplenomegaly or palpable lymphadenopathy. Laboratory values on admission were notable for leukocytosis of 21 × 10^3^/uL (nL ≤ 10 × 10^3^/uL), normal erythrocyte sedimentation rate of 10 mm/hr, elevated C-reactive protein of 2.7 mg/dL (nL < 1 mg/dL), normal thyroid stimulating hormone level of 3.13 u/IU/mL, and a normal lactate dehydrogenase level of 121. He tested negative for HIV. Flow cytometry studies performed on peripheral blood did not reveal any abnormalities.

**Figure 1:**
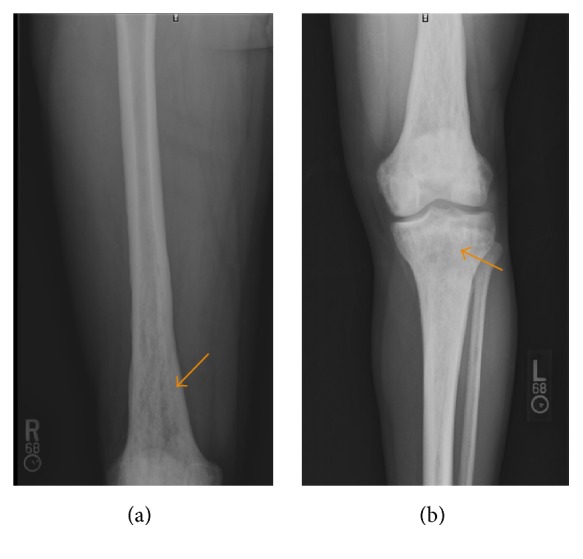
A 35-year-old male with frontal radiographs of the distal right femur and left knee: there is permeative mixed sclerosis and lucency (arrow) in the distal femoral and proximal tibial shafts.

**Figure 2 fig2:**
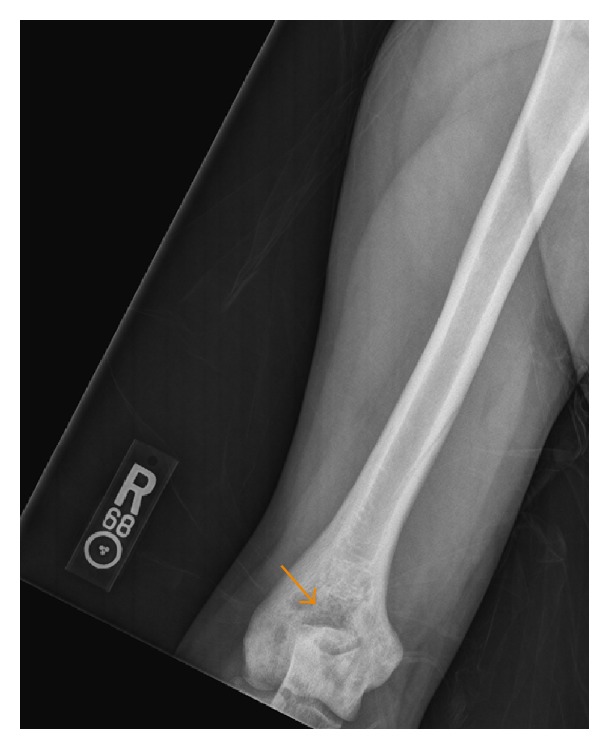
A 35-year-old male with frontal radiograph of the distal right humerus: there is mixed sclerosis and lucency in the distal metaphysis and epiphysis (arrow). Similar findings were present on the contralateral side.

**Figure 3:**
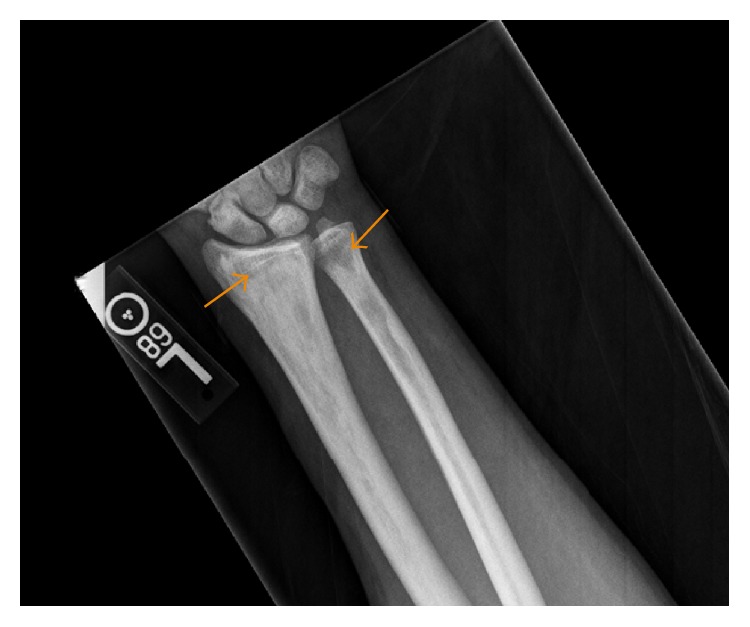
A 35-year-old male with frontal radiograph of the distal right forearm: there is permeative mixed sclerosis and lucency in the distal radial and ulnar metaphysis and epiphysis (arrows). Similar findings were present on the contralateral side.

**Figure 4:**
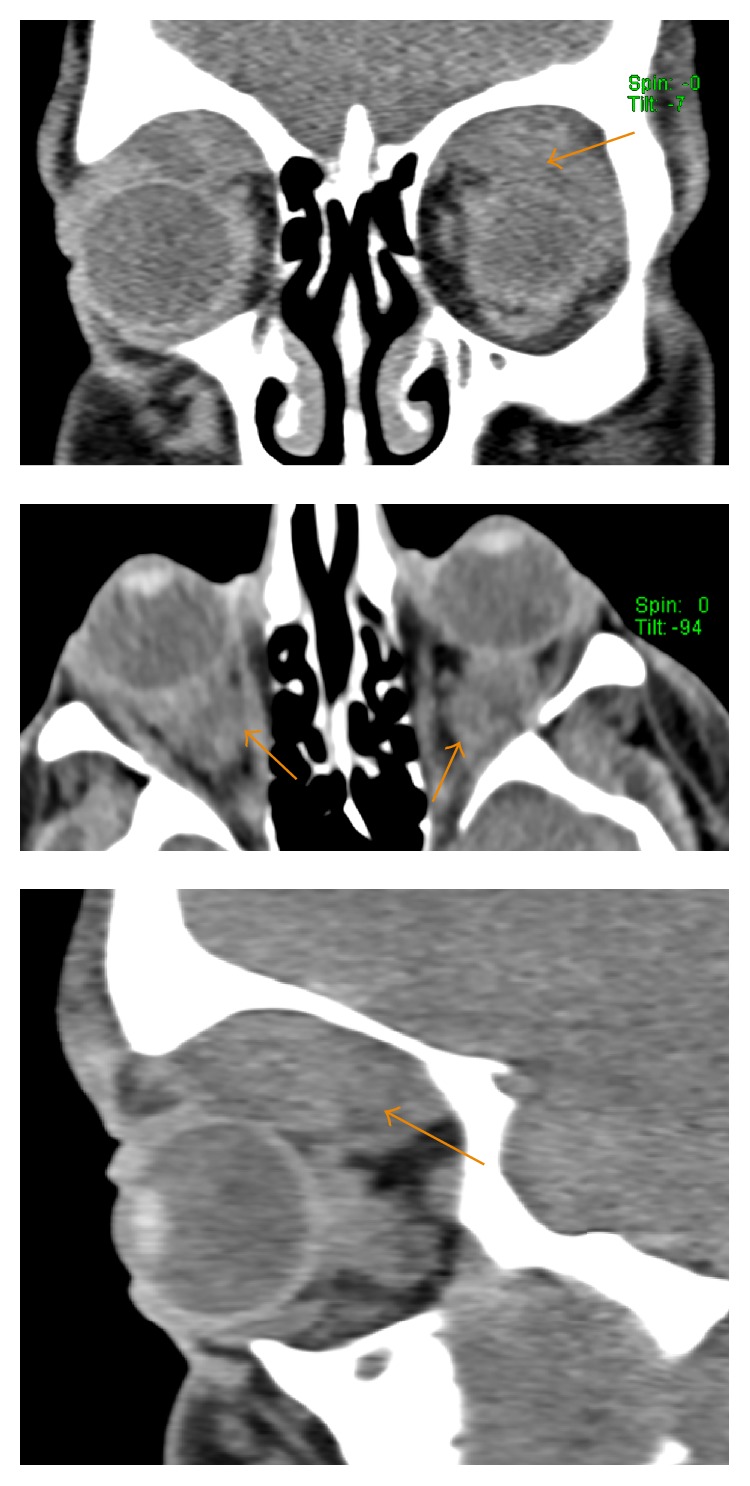
A 35-year-old male with unenhanced axial, coronal, and sagittal reformatted CT images of the orbit: mass-like soft tissue lesions in the intra- and extraconal retrobulbar space (arrow).

**Figure 5:**
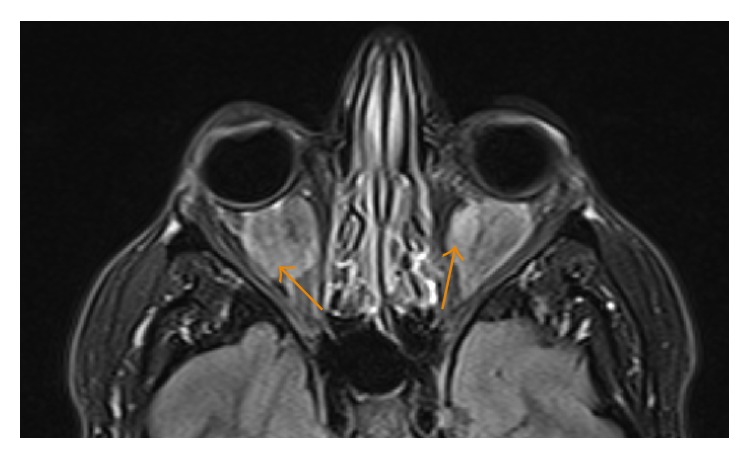
A 35-year-old male with axial T2-weighted FLAIR image showing heterogeneously hyperintense soft tissue lesions (arrow) in the intra- and extraconal retrobulbar space.

**Figure 6 fig6:**
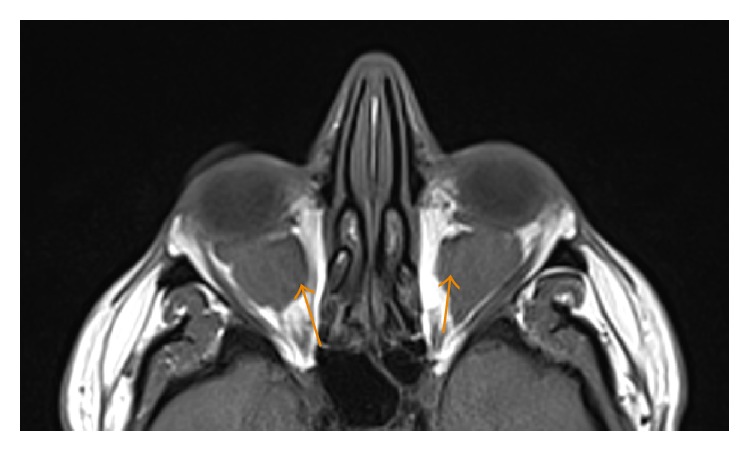
A 35-year-old male with unenhanced axial T1 image showing bulky, homogeneously hypointense soft tissue lesions (arrows) in the intra- and extraconal retrobulbar space.

**Figure 7:**
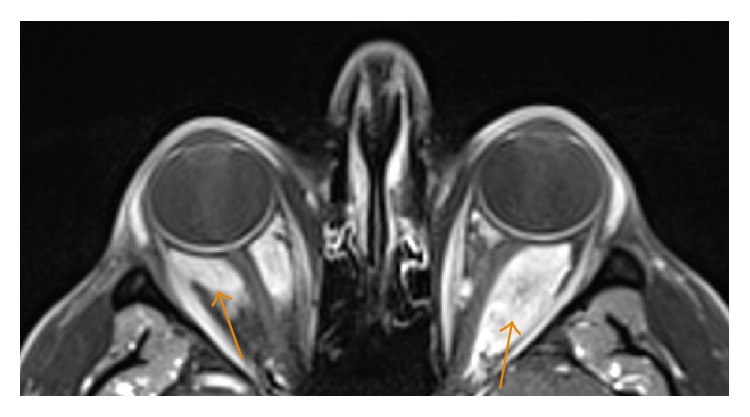
A 35-year-old male with contrast-enhanced, fat-suppressed axial T1 image showing homogeneous enhancement of soft tissue lesions (arrows) in the retrobulbar space which encases the optic nerves bilaterally. The globes are unremarkable. The optic nerves are normal in signal and without enhancement.

**Figure 8:**
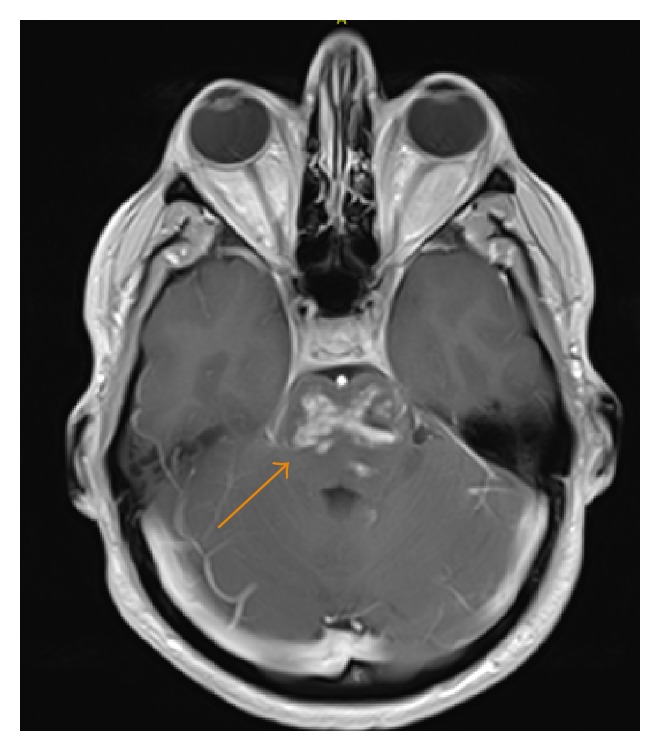
A 35-year-old male with contrast-enhanced axial T1 image showing an expansile heterogeneous patchy enhancement within the pons (arrow).

**Figure 9:**
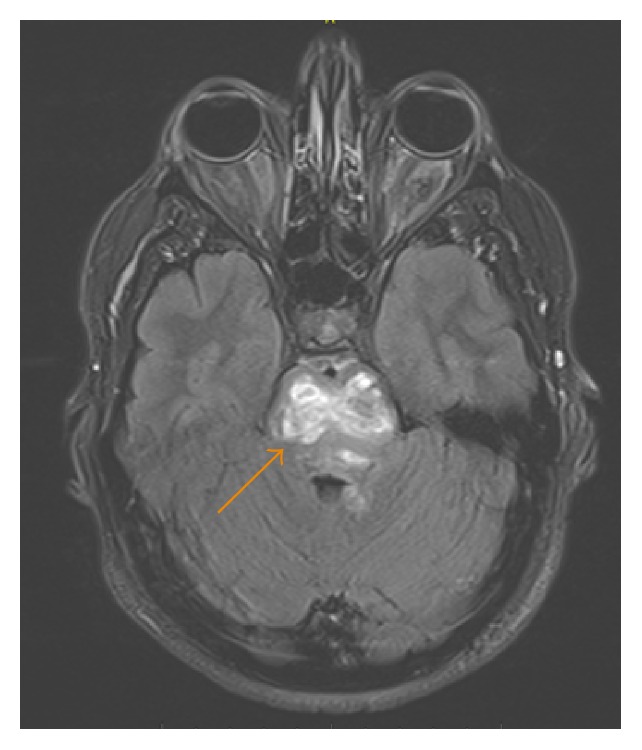
A 35-year-old male with axial T2-weighted FLAI image showing heterogeneous, predominantly hyperintense signal in the pons (arrow).

**Figure 10:**
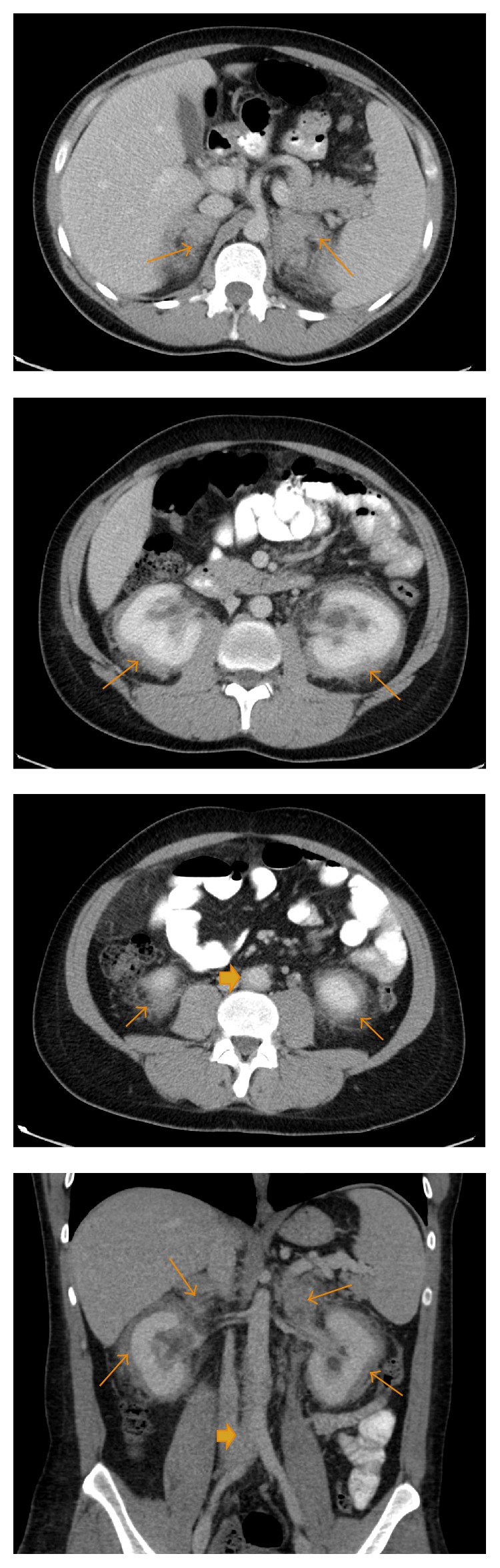
A 35-year-old male with axial and coronal reformatted contrast-enhanced CT images of the abdomen and pelvis showing enhancing soft tissue infiltration of the adrenal glands and kidneys (hairy kidney appearance) bilaterally (thin arrows). This is causing constriction of the renal pelvis and proximal ureters resulting in hydronephrosis. There is also retroperitoneal soft tissue infiltration (thick arrow) covering the posterolateral aorta (coated aorta appearance) which extends from just below level of renal arteries to aortic bifurcation and proximal right common iliac artery.

**Figure 11:**
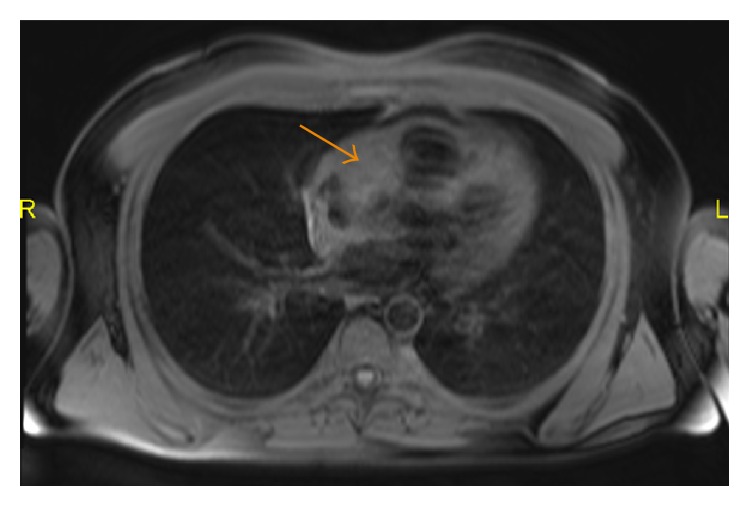
A 35-year-old male with fat-saturated T1-weighted image showing a lesion abutting the right atrium that is isointense to the surrounding muscle.

**Figure 12:**
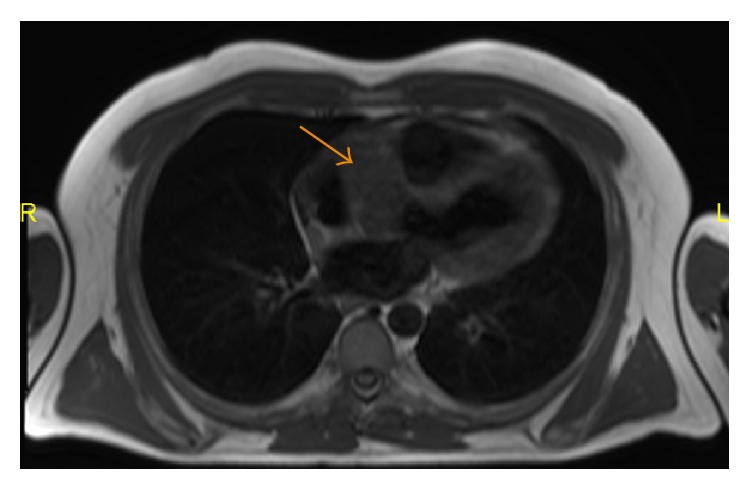
A 35-year-old male with non-fat-saturated T2-weighted image showing a lesion (arrow) abutting the right atrium that is hyperintense to the surrounding muscle.

The patient was initially started on intravenous antibiotics for suspected preseptal cellulitis of the orbit. He subsequently underwent a CT scan of the abdomen and pelvis with oral contrast (OMNIPAQUE 300 milliliters), a radiographic bone survey, and a contrast (MAGNEVIST (Gadopentetate Dimeglumine) 15 milliliters) enhanced MR of the brain and orbits. The radiographic metastatic survey yielded diffuse and bilateral appendicular permeative lucencies mixed with sclerosis (Figures 1 [Fig fig2]–3). The CT of the orbits revealed bilateral soft tissue mass-like lesions in the retrobulbar orbits involving intraconal and extraconal compartments (Figure 4). On MR imaging, these masses were heterogeneously hyperintense on T2-weighted imaging, homogeneously hypointense on T1-weighted imaging, with homogeneous enhancement on postcontrast imaging (Figures 5 [Fig fig6]–7). An expansile mass-like lesion in the pons was also noted which was heterogeneously hyperintense on T2-weighed imaging and demonstrated heterogeneous enhancement on contrast administration (Figures 8 and 9). The contrast-enhanced CT of the abdomen demonstrated a rind of soft tissue covering the lateral and posterior margins of the aorta extending from below the level of the renal arteries down to the aortic bifurcation and proximal right common iliac artery (Figure 10). This classically gave an appearance of a “coated aorta.” Additionally, a ring of enhancing soft tissue surrounding the bilateral kidneys, extending towards the renal sinus and constricting the proximal ureters and renal pelvises, was also seen on the CT abdomen giving the classic appearance of a “hairy kidney” (Figure 10). Lastly, as part of the workup of ECD, a cardiac MR was performed that revealed a small pericardial effusion with a heterogeneously enhancing soft tissue mass measuring approximately 3.6 × 3.4 cm abutting the right atrium (Figures 11 and 12). The patient subsequently underwent a CT guided bone biopsy of a right distal metaphyseal lesion which yielded sclerotic lamellar bone with marrow fibrosis and histiocytic infiltration consistent with ECD.

## 3. Discussion

ECD is a rare multiorgan disease that almost invariably requires tissue sampling for definitive diagnosis. The goal of this report is to highlight those characteristic radiologic findings that are pathognomonic for ECD. Tissue biopsy, though, is still mandatory for definitive diagnosis. Reportedly, as many as 96% of individuals diagnosed with ECD will have skeletal involvement; however only 50% are symptomatic [10]. The most frequently affected bones are the femur, tibia, and fibula and less frequently the ulna, radius, and humerus with pain usually manifesting around the knees and ankles. The hallmark feature, as was the case in our patient, is symmetric osteosclerosis in the diametaphyseal regions of the long bones with relative sparing of the axial skeleton and epiphyseal regions [11]. The radiographic osseous differential diagnosis in this case would include and is not limited to bony lymphoma, Paget's disease, osteomyelitis, and metastases.

Additionally, ECD can also involve the central nervous system including meninges, the orbits, and facial bones and manifest in a wide array of symptoms such as diabetes insipidus, exophthalmos, cerebellar ataxia, panhypopituitarism, and papilledema [12]. In our patient, the presenting symptom was of “bulging eyes” that was misinterpreted to be preseptal cellulitis not responding to antibiotics. CT of the orbits revealed soft tissue mass-like lesions in the retrobulbar orbits that on MRI were shown to be heterogeneously hyperintense on T2-weighted imaging, homogeneously hypointense on T1-weighted imaging, and homogeneously enhancing on postcontrast imaging. All of these findings are consistent with the diagnosis of ECD. In some cases, mass effect of the retroorbital lesions may result in thickening and tortuosity of the optic nerves; however that was not the case in our patient. The differential again is wide and includes but is not limited to Wegener's granulomatosis, Graves' disease, Langerhans cell histiocytosis, lymphoma, sarcoidosis, and Sjogren's disease [12]. There was also evidence of cerebellar involvement in our patient in the form of an expansile pontine lesion which supported his developing symptoms of ataxia. Reportedly, neurological involvement is a prominent feature of ECD, occurring in approximately 51% of the patients over the course of the disease and 23% at disease onset, and an independent predictor of death [13].

Two-thirds of patients with ECD also have evidence of retroperitoneal involvement [14] which is usually an incidental finding. In our patient, there was involvement of the abdominal aorta extending from below the level of the renal arteries down to the aortic bifurcation and proximal right common iliac artery giving the classic appearance of a “coated aorta” [2]. There was also renal involvement resulting in progressive renal failure as the infiltrative tissue compressed on the renal pelvis and subsequently fibrosed around the ureters. This classically is known as the “hairy kidney” appearance which is seen on iodinated CT contrast as the infiltrative process extends into the perirenal fat giving an appearance of an irregular renal border [15]. There was also evidence of cardiac involvement in our patient in the form of a heterogeneous soft tissue mass abutting the right atrium. Cardiac involvement of ECD confers a poor prognosis related to a suboptimal response to treatment [16]. Reportedly, 60% of the 75% suffering from cardiac involvement die from cardiac complications [17] such as congestive heart failure, valvular disease, myocardial infarction, thromboembolism, and cardiac remodeling. Pericardial involvement usually manifests at first in the form of an effusion which is followed by myocardial involvement particularly of the right atrium [18]. The only finding that our patient did not have on presentation was diabetes insipidus that according to Cavalli et al. is one of the more common manifestations of ECD [13].

ECD continues to be a rare form of infiltrative disease but one that is associated with high morbidity and mortality. Timely diagnosis with immediate treatment of this disease remains paramount in achieving some success in countering the progression of this disease. Since most patients will present with nonspecific symptoms of bone pain or eye redness and may subsequently get imaged, one needs to be familiar with the key radiologic findings that may point one into considering ECD in the differential.

## References

[1] Chester W. (1930). Über Lipoidgranulomatose. *Virchows Archiv für Pathologische Anatomie und Physiologie und für Klinische Medizin*.

[2] Diamond E. L., Dagna L., Hyman D. M. (2014). Consensus guidelines for the diagnosis and clinical management of Erdheim-Chester disease. *Blood*.

[3] Jaffe H. L. (1970). *Metabolic, Degenerative and Inflammatory Diseases of Bones and Joints*.

[4] Carpinteri R., Patelli I., Casanueva F. F., Giustina A. (2009). Pituitary tumours: inflammatory and granulomatous expansive lesions of the pituitary. *Best Practice & Research Clinical Endocrinology & Metabolism*.

[5] Volpicelli E. R., Doyle L., Annes J. P. (2011). Erdheim-Chester disease presenting with cutaneous involvement: a case report and literature review. *Journal of Cutaneous Pathology*.

[6] Veyssier-Belot C., Cacoub P., Caparros-Lefebvre D. (1996). Erdheim-Chester disease. Clinical and radiologic characteristics of 59 cases. *Medicine*.

[7] Haroche J., Amoura Z., Trad S. G. (2006). Variability in the efficacy of interferon-*α* in Erdheim-Chester disease by patient and site of involvement: results in eight patients. *Arthritis and Rheumatism*.

[8] Sanchez J. E., Mora C., Macia M., Navarro J. F. (2010). Erdheim-Chester disease as cause of end-stage renal failure: a case report and review of the literature. *International Urology and Nephrology*.

[9] Arnaud L., Pierre I., Beigelman-Aubry C. (2010). Pulmonary involvement in Erdheim-Chester disease: a single-center study of thirty-four patients and a review of the literature. *Arthritis and Rheumatism*.

[10] Haroche J., Arnaud L., Amoura Z. (2012). Erdheim-Chester disease. *Current Opinion in Rheumatology*.

[11] Murray D., Marshall M., England E., Mander J., Chakera T. M. H. (2001). Erdheim-chester disease. *Clinical Radiology*.

[12] Drier A., Haroche J., Savatovsky J. (2010). Cerebral, facial, and orbital involvement in Erdheim-Chester disease: CT and MR imaging findings. *Radiology*.

[13] Cavalli G., Guglielmi B., Berti A., Campochiaro C., Sabbadini M. G., Dagna L. (2013). The multifaceted clinical presentations and manifestations of Erdheim-Chester disease: comprehensive review of the literature and of 10 new cases. *Annals of the Rheumatic Diseases*.

[14] Arnaud L., Hervier B., Néel A. (2011). CNS involvement and treatment with interferon-*α* are independent prognostic factors in Erdheim-Chester disease: a multicenter survival analysis of 53 patients. *Blood*.

[15] Dion E., Graef C., Haroche J. (2004). Imaging of thoracoabdominal involvement in Erdheim-Chester disease. *American Journal of Roentgenology*.

[16] Haroche J., Amoura Z., Charlotte F. (2008). Imatinib mesylate for platelet-derived growth factor receptor-beta positive Erdheim-Chester histiocytosis. *Blood*.

[17] Alharthi M. S., Calleja A., Panse P. (2010). Multimodality imaging showing complete cardiovascular involvement by Erdheim-Chester disease. *European Journal of Echocardiography*.

[18] Haroche J., Cluzel P., Toledano D. (2009). Images in cardiovascular medicine. Cardiac involvement in erdheim-chester disease: magnetic resonance and computed tomographic scan imaging in a monocentric series of 37 patients. *Circulation*.

